# Evaluation of the Stability of Open-Tray Impression Coping Using Two Different Impression Materials at Three Different Subgingival Implant Placement Depths

**DOI:** 10.7759/cureus.61117

**Published:** 2024-05-26

**Authors:** Gayathri P.M, Kala Sukumaran, Harshakumar K, Smitha Ravindran

**Affiliations:** 1 Prosthodontics, Government Dental College, Thiruvananthapuram, Thiruvananthapuram, IND

**Keywords:** dental implantology, dental impression technique, impression, implant final impression, dental titanium implant

## Abstract

Objective

To evaluate and compare the stability of the open tray impression coping within the set impression while attaching the lab analog when polyether (PE) heavy body and polyvinyl siloxane (PVS) putty impression materials were used and the implant platform was placed sub-gingivally at three different depths.

Methods

Two impression materials, PE and PVS, and custom-made plexiglass models with embedded single implants to simulate implant positioning depths of 0 mm, 2 mm, and 4 mm, sub-gingivally, were used in the study. Open tray impressions were made after attaching impression coping to the implant embedded in the model. Implant lab analog was attached to the impression coping in the set impression, and its stability was measured using a universal testing machine. Forty-two open tray impressions were made in six groups, with seven impressions in each group. Descriptive statistics such as mean and standard deviation were calculated. A comparison of the mean stability between the two impression materials at each depth was done using an independent t-test. Comparison of the mean stability between the three different subgingival implant depths in each material was done by one-way ANOVA with the Scheffe multiple comparison test (post-hoc analysis). The level of significance was set at p<0.05.

Results

The stability of the impression coping was measured as the force in Newtons required for the displacement of the analog attached to the impression coping embedded in the set impression. PE with the embedded impression coping at a depth of 0 mm gave the highest mean stability value (4.37+/-0.41), and the least mean stability was offered by PVS with the embedded impression coping at 4 mm depth (1.88+/-0.37). When an independent t-test was done to compare the mean stability values of PE and PVS, there was a statistically significant difference at 0 mm, 2 mm, and 4 mm. On doing one-way ANOVA to compare the mean stability between the different depth groups, there was a statistically significant difference between the three depth groups in PE and PVS. Scheffe multiple comparison tests (post-hoc analysis) revealed a statistically significant difference between 0 mm, 2 mm, and 4 mm subgingival depths of the impression coping placement in both PE and PVS.

Conclusion

The accuracy of the master cast is an important determinant for the precise fit and long life of the final prosthesis. In the case of maxillary anterior implant placements where deep subgingival placement of the implant platform is needed for aesthetic and functional reasons, the impression material should be selected carefully to ensure the stability of the impression coping. Among the materials included in the present study, the PE impression material offered the maximum stability for impression coping compared to PVS.

## Introduction

The stability of an impression coping embedded in the set impression is the resistance against displacement while attaching the lab analog. Impression coping is a component of a dental implant system used to provide a spatial relationship of the endosteal dental implant to the alveolar ridge and adjacent dentition or other structures [1]. There are three types of impression techniques, closed-tray technique (indirect technique), open-tray technique (direct technique), and digital technique [2]. In the open-tray technique, impression coping and a modified custom tray with a window corresponding to the implant position are used for impression-making. It is the most accurate technique for multiple implants and implants placed sub-gingivally [3].

To achieve a highly aesthetic and long-lasting prosthesis, certain guidelines should be followed for the three-dimensional positioning of implants [4]. The implant should be positioned at a depth of 3 mm from the cervical contour of the planned crown to achieve the appropriate biological width [5,6]. After implant placement, the pick-up impression coping is tightened on the implant in the oral cavity with screws, and the fit of the implant-coping junction is verified radiographically. After making the impression, a lab analog is attached to the impression coping to represent the implant in the master cast and tightened. During the attachment and tightening of the implant lab analog on the coping embedded in the set impression in the tray, there are chances of displacement of the impression coping. This may result in an inaccurate master cast and, finally, an inaccurate final prosthesis leading to screw loosening, bending, and fracture of the prosthesis or implant components, occlusal inaccuracies, marginal discrepancies, and bone loss [7]. Thus, the embedded impression coping should resist the displacing forces while attaching the analog.

While making the impression of multiple implants by following the open-tray technique, the implants can be splinted together to resist horizontal displacement and to make them stable [8]. Splinting is not possible for making an impression in a deeply placed single dental implant. There are chances of horizontal displacement during the procedure of positioning the lab analog on the impression coping. The use of longer impression copings for subgingival implants has been suggested to overcome this problem. However, only a few implant systems offer this solution, and it is not possible to use in patients with restricted mouth opening [9]. Kim et al. conducted a study on the conventional open-tray impression versus intraoral digital scans for implant-level complete-arch impression and found that conventional open-tray impressions produced significantly smaller linear displacements than digital scans obtained using an intraoral scanner at the implant level in a complete-arch model [10]. Yasar et al. conducted a review of the implant impression techniques using different materials and methods and concluded that open-tray impressions and digital impressions produced more accurate results when compared to other techniques. They also concluded that either polyvinyl siloxane (PVS) or polyether (PE) can be used for making impressions with minimum errors [2]. The digital impression technique is not economically feasible in all situations. Papaspyridakos et al. conducted a systematic review in 2014 on the accuracy of implant impressions and concluded that PVS and PE have no effect on the accuracy of the impression [11]. Conrad et al. investigated the accuracy of two impression techniques with angulated implants and reported that, if the impression coping is not stable, it may result in the rotation of impression coping while connecting the dental implant analog [12]. The present study was conducted to find out the most suitable material among PE and PVS that can be used for deeply placed dental implants that provide maximum stability for impression coping during open-tray implant-level impression procedures.

## Materials and methods

A protocol was submitted before the Institutional Ethics Committee, Government Dental College, Thiruvananthapuram, India, to conduct an in-vitro study on the stability of open-tray impression coping using two different impression materials at three different subgingival implant placement depths, and permission was obtained (Ref. no. DCT/IEC/E/03/2017).

The study aimed to determine the stability of open-tray impression coping while using PE and PVS impression materials at 0 mm, 2 mm, and 4 mm subgingival implant placement depths. The study was conducted by keeping two null hypotheses. The first null hypothesis was that there was no statistically significant difference in the stability of open-tray impression coping when the impression was made with PE and PVS. The second null hypothesis was that there was no statistically significant difference in the stability of open-tray impression coping when the impression was made at 0 mm, 2 mm, and 4 mm subgingival implant placement depths.

**Figure 1:**
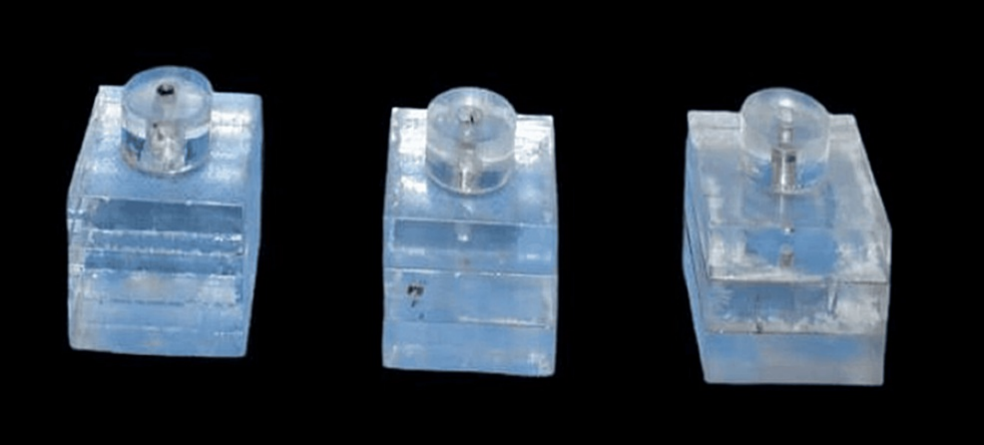
Plexiglass models embedded with implants at 0 mm, 2 mm, and 4 mm to represent the different subgingival depths in the study.

Three plexi glass-based models were fabricated for the study similar to the in vitro study conducted by Linkevicius et al. [13]. Implant platform depths of 0 mm, 2 mm, and 4 mm below the gingival surface were created by placing one implant per model (Figure 1). Implants with internal hex were used in the study to represent the most commonly used implant type and to avoid confounding factors that affect the stability of impression coping. A hole was drilled using a micromotor in the center of each model sufficient to accommodate a single dental implant (Genesis Aktiv, JJ implants, Thrissur, Kerala, India) of 3.75 mm diameter and 11.5 mm length, and the implant was fixed with an adhesive. In the first model, the implant was embedded to represent an implant platform depth at 0 mm sub-gingivally. In the second model, the implant with the same dimension was embedded 2 mm deep to represent an implant platform depth of 2 mm sub-gingivally. In the third model, a similar implant was placed to represent an implant platform depth of 4 mm sub-gingivally. An open-tray impression coping was connected to each dental implant, and the guide pin was hand-tightened.

**Figure 2:**
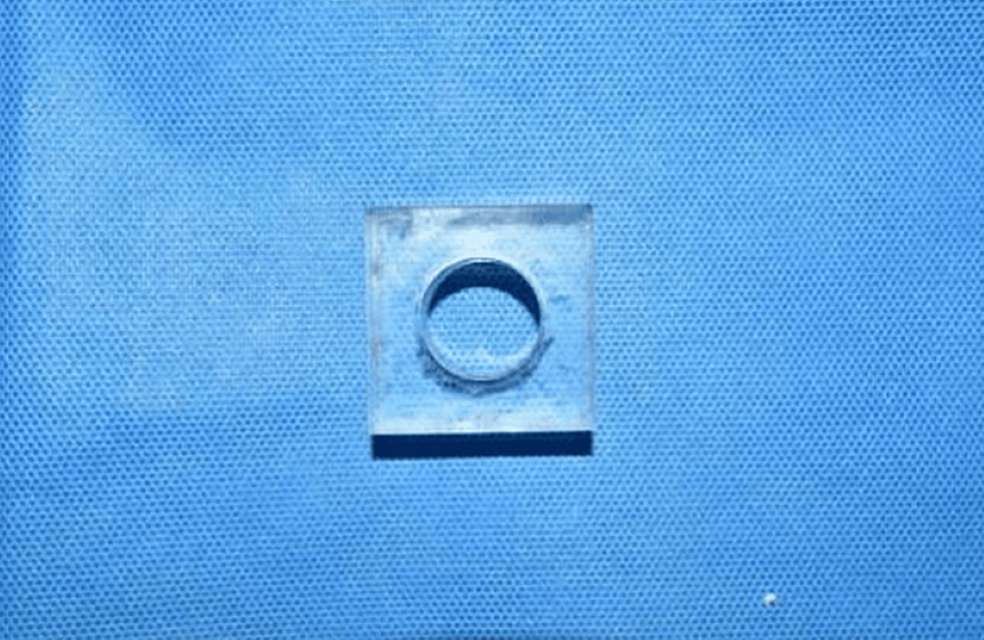
Plexiglass impression tray with an opening in the centre for making the open-tray impression.

Custom impression trays were fabricated with plexiglass with an opening in the centre (Figure 2), similar to the impression trays used in the open impression technique. The impression materials used in the study were PE heavy body consistency (Permadyne, 3M, India) and PVS putty consistency (Flexceed, GC corporation, India). All the materials were manipulated according to the manufacturer’s instructions. The respective tray adhesive was applied before loading the tray with impression material. The trays loaded with impression material were used to make the impression of the models with the attached impression coping. After the impression was set, the guide pin was removed, the impression was separated from the model, and the lab analog was attached to each impression coping embedded in the set impression using the guide pin.

**Figure 3:**
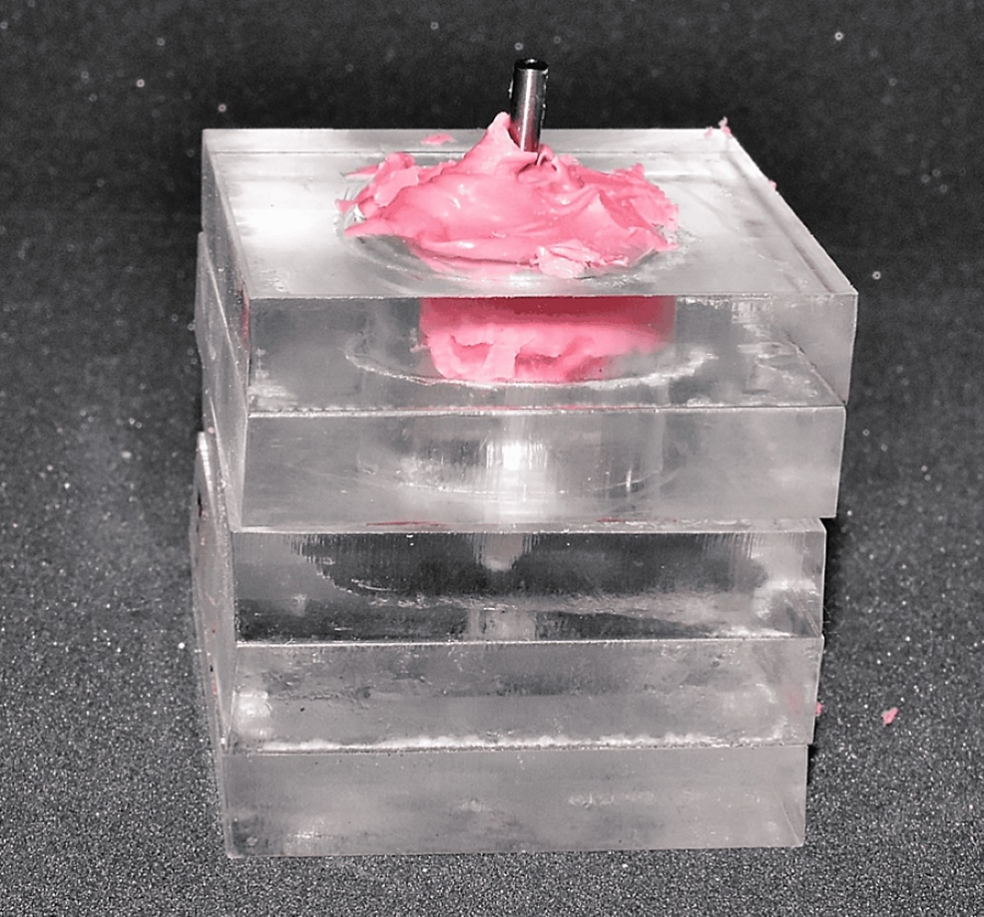
Impression made with a polyether impression material.

**Figure 4:**
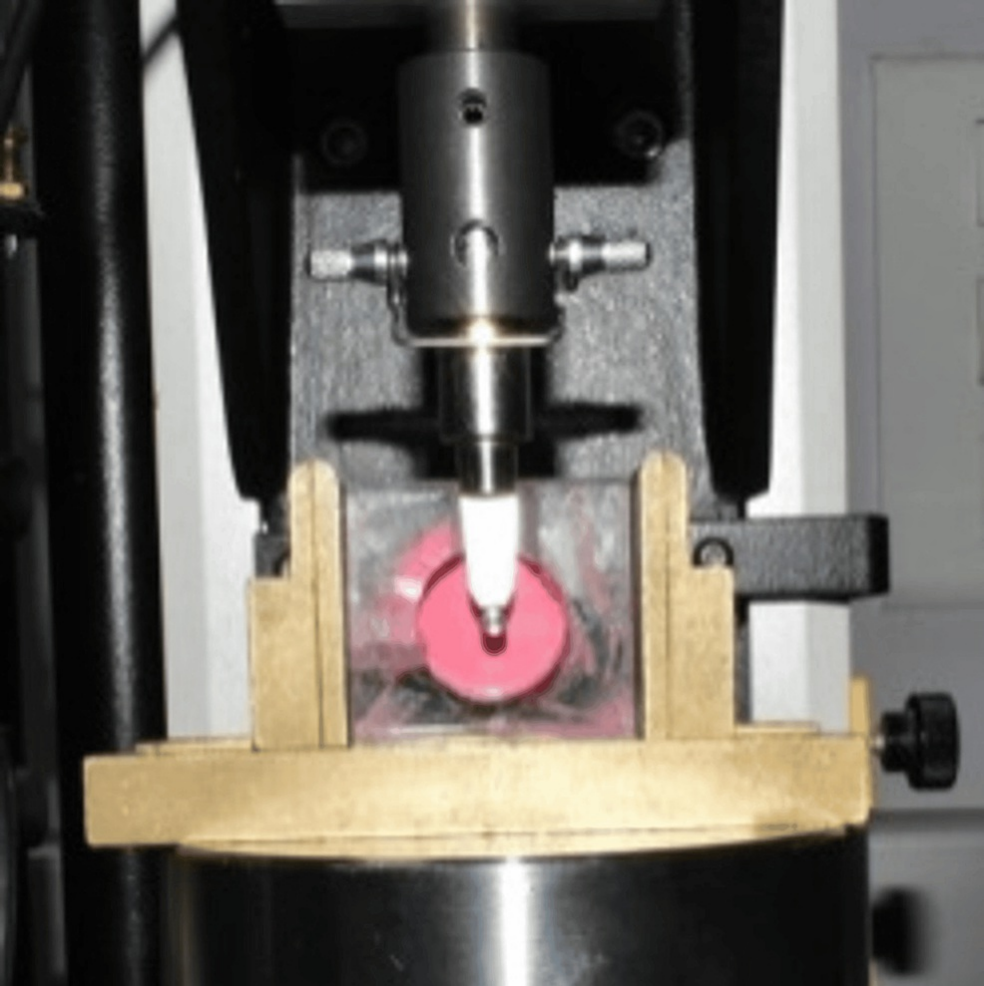
Measurement of the stability of implant coping in polyether impression.

There were six groups of impressions in the study. In the first three groups, the impression was made with PE (Figure 3) at three depths: 0 mm (Group A1), 2 mm (Group A2), and 4 mm (Group A3). In the next three groups, the impression was made with PVS at three depths: 0 mm (Group B1), 2 mm (Group B2), and 4 mm (Group B3). Seven impressions were made in each group, and a total of 42 impressions were made. After making the impressions, the implant lab analog was connected to the open-tray impression coping, the guide pins were hand-tightened without pressure, and the impressions were stored at room temperature for a minimum of 12 hours before the measurement was done to mimic changes in the material for an average time delay during the transportation for the preparation of master cast. A universal testing machine (Instron model 3345; Instron, Norwood, MA) was used to test the stability of impression coping. The plexiglass tray with impression was placed in a locking device, and a knife-edge metallic tool attached to the universal testing machine was made to touch the surface of the lab analog connected to the coping (Figure 4). A compression test was done with a crosshead speed of 1 mm/min and a 100 N load cell for measurement [13]. Load at 1 mm compressive extension was determined. The machine was programmed such that the tip of the instrument would stop after moving the lab analog connected to the impression coping by 1 mm. The force obtained in Newton was recorded. A single operator carried out all the laboratory procedures. The mechanical testing was performed similarly for all the groups, and the data obtained were recorded.

The data collected were entered in Microsoft Excel. Statistical analysis was done using SPSS software (version 25.0; IBM Corp., Armonk, NY). Shapiro-Wilk and Kolmogorov-Smirnov tests were done to test the normality of the data. Descriptive statistics were expressed in terms of mean and standard deviation. An independent t-test was used to compare the mean stability of the impression coping embedded in PVS and PE at each depth. One-way ANOVA, followed by Scheffe multiple comparison tests (post-hoc analysis), was done to compare the mean stability between the three subgingival depths in each material. The level of significance was set at p<0.05.

## Results

The normality of the obtained data was assessed using the Shapiro-Wilk test for each group, and it was observed that the distribution was normal (p > 0.05). The study results are given in Table 1. The impression coping embedded in the PE impression material at 0 mm subgingival depth gave the maximum mean stability, and the coping embedded in the PVS impression at 4 mm subgingival depth gave the least mean stability. While comparing 0 mm, 2 mm, and 4 mm subgingival depths, the highest mean stability was observed at 0 mm subgingival depth for both impression materials.

**Table 1:** Descriptive statistics showing the stability of open-tray impression coping between PE and PVS at 0 mm, 2 mm, and 4 mm subgingival depths. SD - Standard deviation; PE - Polyether; PVS - Polyvinyl siloxane

		PE	PVS
0 mm	Mean +/- SD	4.37+/-0.41	3.2+/-0.29
	Median	4.15 (4.02-4.87)	3.11 (2.97-3.57)
	Minimum	4.01	2.90
	Maximum	4.95	3.63
2 mm	Mean +/- SD	3.31+/-0.15	2.59+/-0.17
	Median	3.26 (3.24-3.98)	2.65 (2.43-2.71)
	Minimum	3.04	2.29
	Maximum	3.48	2.78
4 mm	Mean +/- SD	2.58+/-0.37	1.88+/-0.37
	Median	2.6 (2.17-2.94)	1.92 (1.71-2.04)
	Minimum	2.08	1.22
	Maximum	2.94	2.46

A comparison of the mean stability at each depth between PE and PVS, and the results of the independent t-test are given in Table 2. Among PE and PVS at 0 mm, PE gave a high stability value when compared to PVS. An independent t-test between PVS and PE at 0 mm (t-value = 6.14, p < 0.01) found that there was a statistically significant difference in stability between the copings embedded at 0 mm in PE and PVS. At a subgingival implant placement depth of 2 mm, PE offered higher mean stability among the two impression materials. On doing an independent t-test between PE and PVS at 2 mm, a statistically significant difference in stability (t = 8.28, p < 0.01) between the copings was found. Among PE and PVS at a subgingival implant placement depth of 4 mm, more stability of the impression coping was provided by PE. An independent t-test between PVS and PE at 4 mm revealed a statistically significant difference in stability (t = 3.53, p < 0.01) between the copings.

**Table 2:** Statistical analysis of the stability of open-tray impression coping between PE and PVS at depths 0 mm, 2 mm, and 4 mm. Results of the independent t-test. N - Number of samples; p - Level of significance The level of significance was set at p < 0.05.

		Mean	SD	N	t	p
0 mm	PE	4.4	0.4	7	6.14	p<0.01
	PVS	3.2	0.3	7		
2 mm	PE	3.31	0.15	7	8.28	p<0.01
	PVS	2.59	0.17	7		
4 mm	PE	2.58	0.37	7	3.53	p=0.04
	PVS	1.88	0.37	7		

A comparison of the stability of open-tray impression coping was done between the three depth groups using one-way ANOVA and Scheffe multiple comparison (post-hoc test) when PE and PVS were used. The results are given in Table 3. On comparing the mean stability values between the depths, there was a statistically significant difference in the mean stability between 0 mm, 2 mm, and 4 mm while using each impression material. On comparison between the different groups of subgingival positioning depth, a statistically significant difference in the stability of impression coping was observed between all the three groups of subgingival implant placement depths in PE and in PVS impression materials.

**Table 3:** Statistical analysis of open-tray impression coping among different depths for PE and PVS impression materials. Analysis using one-way ANOVA and Scheffe multiple comparisons (post-hoc test) SD - Standard deviation; F - Ratio of the variables between multiple samples The level of significance was set at p < 0.05.

Impression material	Depth	Mean	SD	N	F	p	Scheffe multiple comparison		
							Pair	F	P
PE	0 mm (A1)	4.37	0.41	7	51.93	p<0.01	A1&A2	18.1	P<0.01
	2 mm (A2)	3.31	0.15	7			A1&A3	51.3	P<0.01
	4 mm (A3)	2.58	0.37	7			A2&A3	8.5	0.003
PVS	0 mm (B1)	3.20	0.29	7	36.38	p<0.01	B1&B2	7.8	P<0.01
	2 mm (B2)	2.59	0.17	7			B1&B3	36.3	P<0.01
	4 mm (B3)	1.88	0.37	7			B2&B3	10.4	P<0.01

## Discussion

Dental Implantology is a prosthetically driven science, where an accurate impression is the primary foundation for successful reconstruction [14]. The accuracy of impression is influenced by various factors, such as depth, angulation and position of implants, impression materials, impression technique, type of impression trays, level of impression (implant/abutment level), design of impression coping, splinting or non-splinting of impression coping, and time delay for impression pouring [15]. The results of the study showed that, in deeply placed subgingival implants, the PE impression material provided statistically significant stability to impression coping compared to the PVS impression material.

There are three components of peri-implant soft-tissue phenotype: keratinized mucosa width (KMW), mucosal thickness (MT), and supracrestal tissue height (STH) [16]. These three components of peri-implant soft tissue play a key role in the stability of the peri-implant marginal bone tissue [17]. Research has shown that implants placed in a site with short STH (< 2 mm) exhibit more marginal bone loss due to physiologic STH establishment [18,19]. Hermann et al. [20] suggested that a stable biological width is essential to maintain the hard and soft tissue health around the implant and to prevent gingival recession. According to Kan et al. [6], the average dimension of biological width in relation to implant is 3 mm. Cochran et al. have reported that the biological width is 3.3 mm for dental implants, including the sulcus depth [21]. Thus, the implant should be positioned at 3-3.3 mm from the cervical contour of the planned crown to achieve appropriate biological width [5,6]. If the bone level is more than 3 mm in the apical direction from the cervical contour, a bone grafting procedure is indicated, and the bone needs to be reduced to create space for biological width if the bone level is less than 3 mm [4,5,22]. Subcrestal implant placement is a clinical strategy to reduce the risk of the crestal part of the implant becoming exposed by the early marginal bone loss [17] and to promote increased STH in the second stage of surgery [23]. Thus, during the implant-level impression procedure, the subgingival depth for registering the impression is about 4 mm.

During the implant-level impression procedure using an open tray, an impression coping is attached directly to the implant to transfer the three-dimensional orientation of the implant to the master cast. The impression material selected for making the impression should provide stability to the coping during the procedure of attaching the analog. Two impression materials, PE and PVS, which are used in implant impressions, were studied to find out the material that offers maximum stability. The subgingival depth of 0 mm was selected in this study to represent the impression procedure at the abutment level. In the case of implant-level impressions, the total subgingival depth of the implant platform is nearly 4 mm. A subgingival depth of 2 mm is selected to keep as a reference for comparing the stability value of a maximum subgingival depth of 4 mm. Thus, 2 mm and 4 mm subgingival depths were included in the present study.

Martinez et al. selected three subgingival implant depths 0, 1, and 3 mm to study the accuracy of the master cast and proved that there was a statistically significant difference in the accuracy when different implant angulations and depths were employed [24]. A study by Aidasani et al. [25] proved that vinylpolysiloxane had the highest degree of stiffness among the impression materials examined in comparison to PE and vinylsiloxanether, as seen by its much greater rotational resistance to torquing.

In the present study, it is proved that the PE heavy body impression material provides a significantly higher impression coping stability during the open impression procedure when compared to the PVS impression material. The results agree with the research by Wee [26] that PE produced the highest overall torque values, followed by the addition silicone and polysulphide. It is also proved in the present study that, as the subgingival depth of the implant is increased, the the stability of the impression coping is decreased. This result is similar to the findings of Linkevicius et al. [13] that there was reduced stability of the impression coping in the polymerized impression material when the level of the implant platform was deep. The findings also agree with Lee et al. [9], who evaluated the effect of subgingival depth of implant placement on the accuracy of implant impressions and proved that, among the PE impressions, 4 mm subgingival depth of implants resulted in greater horizontal distortion compared to implants placed more coronally. Clinically, nearly 4 mm subgingival depth for implant placement is needed for functional and aesthetic reasons. The optimum stability of the impression coping can be achieved by following the open-tray impression technique and by using the PE heavy body impression material for the maximum accuracy of the master cast.

On completion of the study, it was proved that there is a statistically significant difference in the stability of the open-tray impression coping when the impression is made using PE and PVS impression materials. It is also proved that there is a statistically significant difference in the stability of the open-tray impression coping at 0 mm, 2 mm, and 4 mm subgingival implant placement depths. Hence, both the null hypotheses are rejected.

PE offers high tear strength and better resistance to deformation and is hydrophilic in nature. This is the material of choice for the implant impression, to accurately record and transfer the implant position and orientation from the patient’s mouth to the working cast. The only disadvantage with PE is that it cannot be stored for a long time because the dimensions of this material start changing after a few days, which may lead to inaccuracy of the implant prosthesis in the precision of fit.

This study has the following limitations. The evaluation was done in in-vitro settings, and variation in the results of the present study can be anticipated in in-vivo study settings. The newly introduced, vinylsiloxanether, impression material, which possesses the advantages of PVS and PE impression materials, was not considered for this study.

## Conclusions

The subgingival depth of the implant margin and the type of impression materials used have a significant role in the outcome of implant prostheses. The stability of open-tray impression copings plays a crucial role in achieving an accurate master cast. In the comparison of the stability of open-tray impression coping embedded in the pick-up impression while attaching the implant lab analog, using a PE heavy body and PVS putty consistency, it was found that the impression coping was more stable with the PE impression material (heavy body consistency) and that the stability of the open-tray impression coping decreased with an increase in subgingival implant placement depth.

Clinicians must be aware that, as the subgingival level of implant placement is increased, the stability of the open-tray impression coping will decrease. In such cases, the implant depth should be cautiously selected, and an open impression technique with PE heavy body should be used to get the most accurate master cast and a long-lasting implant prosthesis.
